# Myeloid-Derived Suppressor Cells (MDSCs) in Ovarian Cancer—Looking Back and Forward

**DOI:** 10.3390/cells12141912

**Published:** 2023-07-22

**Authors:** Karolina Okła

**Affiliations:** 1 The First Department of Oncologic Gynecology and Gynecology, Medical University of Lublin, 20-081 Lublin, Poland; karolinaokla@umlub.pl or kokla@med.umich.edu; 2 Department of Surgery, University of Michigan, Ann Arbor, MI 48109, USA

**Keywords:** myeloid-derived suppressor cells, MDSCs, ovarian cancer, immunotherapy, CAR-M, TME, therapy resistance

## Abstract

Myeloid-derived suppressor cells (MDSCs) play a significant role in the immune system and have been extensively studied in cancer. MDSCs are a heterogeneous population of myeloid cells that accumulate in the tumor microenvironment. Consequently, the high abundance of these cells often leads to immunosuppression, tumor growth, treatment failure, and poor prognosis. Ovarian cancer ranks fifth in cancer deaths among women, accounting for more deaths than any other cancer of the female genital tract. Currently, there is a lack of effective clinical strategies for the treatment of ovarian cancer. Although several studies underline the negative role of human MDSCs in ovarian cancer, this topic is still understudied. The works on MDSCs are summarized here, along with an explanation of why focusing on these cells would be a promising approach for treating ovarian cancer patients.

## 1. Introduction

More than 15 years ago, scientists coined the term myeloid-derived suppressor cells (MDSCs) [1]. Accumulation of these cells has been reported in pathological conditions including infectious diseases, acute and chronic inflammation, traumatic stress, and cancer. The discovery of three different populations of human MDSCs, including monocytic M-MDSCs, polymorphonuclear PMN-MDSCs, and early-stage eMDSCs, underlines their heterogenous nature, which has been broadly study in many cancer types [2,3,4]. Twelve years ago, Obermajer and colleagues examined MDSCs in human ovarian cancer (OC) for the first time. Overall, these studies opened a new chapter in the MDSC field in OC [5].

OC is the most lethal of all malignancies of the female reproductive system [6]. According to the Global Cancer Observatory’s 2020 projections, by 2040, the number of women around the world diagnosed with OC will rise by almost 42% to 445,721. The number of women dying from OC each year is projected to increase to 313,617—an increase of over 50% from 2020 (Global Cancer Observatory, https://gco.iarc.fr/ (accessed on 10 July 2023)). Because of the insidious symptoms, only 20% of patients can be identified at the early stages [7]. Although patients with OC respond to chemotherapy, the effects are short-lasting. More than 80% of OC patients relapse, and more than 50% of these patients die from the disease in less than 5 years post-diagnosis. Furthermore, patients often develop chemotherapeutic resistance. Importantly, contrary to expectations, clinical trials using immune checkpoint therapy (ICT), i.e., anti-PD-1/PD-L1, have shown a limited response rate of about 10–15%, and there is no current FDA/EMA approval for disease [8]. Poor infiltration by immune cells and active immunosuppression in the tumor microenvironment (TME) make OC insensitive to ICT [9]. In this context, a strongly immunosuppressive TME may considerably contribute to disease progression and metastatic dissemination, calling for the implementation of new immunotherapeutic strategies beyond ICT.

MDSCs are known to contribute to tumor immune evasion and serve as a central component in the immunosuppressive network of many tumors. It has been shown that MDSCs block the recruitment and priming of T cells, resulting in the T cell exclusion phenotype within the TME [10]. Furthermore, MDSC infiltration is associated with treatment failure and poor prognosis in many cancers [11,12]. Recent advances have shown MDSC-mediated PD-1/PD-L1 treatment resistance [13]. Thus, inhibiting MDSCs’ activity may sensitize tumors to ICT and thereby overcome therapeutic resistance. The main characteristics of MDSCs are outlined in this article, with an emphasis on their function in ovarian cancer and how these cells can be exploited in cancer therapy to overcome therapeutic resistance.

## 2. MDSCs in Cancer

**Figure 1:**
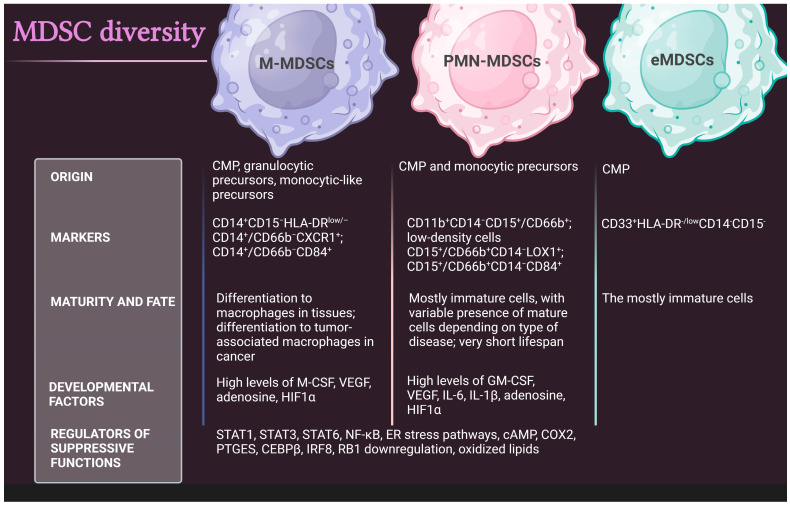
Comparison of the basic characteristics of human MDSC populations. CMP, common myeloid progenitor; eMDSCs, early-stage MDSCs, G-CSF, granulocyte colony-stimulating factor; GM-CSF, granulocyte–macrophage colony-stimulating factor; HIF1α, hypoxia inducible factor 1α; M-CSF, macrophage colony-stimulating factor; MDSC, myeloid-derived suppressor cell; M-MDSC, monocytic MDSC; PMN, polymorphonuclear; PTGES, prostaglandin E synthase; VEGF, vascular endothelial growth factor.

MDSC levels, including M-MDSCs, PMN-MDSCs, and eMDSCs, are negligible in healthy individuals, while their levels increase in cancer [11,14]. Pathological activation arises from persistent stimulation of the myeloid cells owing to the prolonged presence of myeloid growth factors and other inflammatory signals in the TME (e.g., M-CSF, GM-CSF VEGF, HIF1α, and IL-6). The major regulators of suppressive functions of MDSCs include STATs, NF-κβ, cAMP, COX2, and others. The endoplasmic reticulum (ER) stress response signaling, RB1 downregulation, and lipid oxidation have also been implicated in the suppressive activity of MDSCs. The diversity and characteristics of human MDSCs are shown in Figure 1.

**Figure 2:**
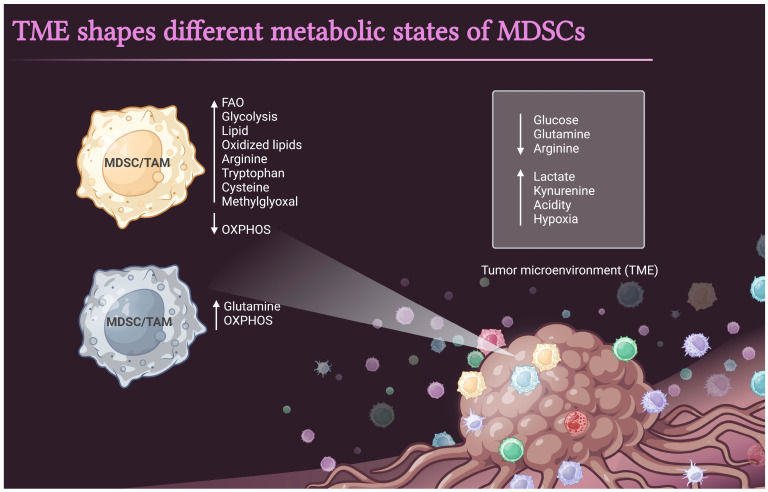
Metabolic characteristics of the MDSCs in the TME. Metabolic changes that occur in the MDSCs and the TME are shown. The MDSCs in the TME exhibit upregulation of fatty acid oxidation and glycolysis and a decrease in oxidative phosphorylation (OXPHOS). MDSCs also show increased lipid accumulation and increased production of metabolites, including arginine, tryptophan, cysteine, and methylglyoxal. In contrast, it has been shown that MDSCs acquire an energetic metabolic phenotype promoted primarily by increased OXPHOS fueled by glutamine. Key changes in the TME are depicted in the right box.

In the TME, competition for nutrients and oxygen forces immune cells to adapt their metabolism. MDSCs sense the changes in the environment and respond by selecting the most efficient metabolic pathways to sustain their suppressive and pro-tumorigenic functions [12,15] (Figure 2). Diets rich in polyunsaturated fatty acids or high-fat diets have been shown to favor the differentiation of MDSCs from bone marrow precursors and to potentiate the suppressive activity of these cells in mice [16]. The upregulation of glycolytic pathways protected MDSCs from apoptosis and contributed to their survival by preventing ROS-mediated apoptosis via the antioxidant activity of the glycolytic intermediate phosphoenolpyruvate [17]. Furthermore, deprivation of essential metabolites, including arginine, cysteine, and tryptophan from the TME, has been used by MDSCs to impair T cell function [12]. Similarly, methylglyoxal has been identified as a marker of MDSCs and may play a key role in the suppression of CD8^+^ T effector function [18]. Due to their great plasticity, MDSCs in vivo can display unique metabolic profiles depending on tissue origin and the TME [15]. Indeed, myeloid cells primed by ID8 ovarian cancer cells showed increased oxidative phosphorylation fueled by glutamine [19]. Collectively, these results indicate the metabolic plasticity of MDSCs.

As a key component of the TME, MDSCs utilize multiple mechanisms to inhibit immune responses and promote tumor progression. On the one hand, MDSCs promote the formation of an immunosuppressive TME, which in turn exerts an influence on the biology and function of MDSCs. On the other hand, MDSCs also enhance tumor progression and induce resistance to antitumoral therapy in different non-immunological manners (Table 1). First, MDSCs can play a critical role in facilitating tumor immune escape by inhibiting cytotoxic T lymphocytes, natural killer (NK) cells, antigen presenting cells (APCs), and B cells via the expression of checkpoint molecules, depleting nutrients, and the induction of oxidative stress in the TME [20]. Second, non-immunological functions of MDSCs including the promotion of angiogenesis, stemness, epithelial–mesenchymal transition (EMT), and metastases of cancer cells further enhance tumor progression [20,21]. Metastasis is responsible for about 90% of cancer deaths [22], and MDSCs are well known for the formation of premetastatic niches in cancer. It has been shown that MDSCs promote metastasis by building the premetastatic niche to enhance the engraftment of circulating tumor cells (CTCs) and by escorting tumor cells into the circulation, which promotes their metastatic potential, inhibits their killing by T cells, and promotes their extravasation into the tissues [12]. As this area of research remains largely understudied, further focusing on the role of MDSCs in the ‘priming’ of the premetastatic niche is needed, and inhibiting the premetastatic niche can be clinically and therapeutically valuable.

## 3. MDSCs in Human Ovarian Cancer

Although much progress has been made in recent years towards studying MDSCs in cancer, only a few works have been published on human OC (Table 2).

The first report on MDSCs in ovarian cancer patients was published in 2011, and the authors showed that in ascites isolated from patients, both CXCL12 and CXCR4 are controlled by the tumor-associated inflammatory mediator prostaglandin E2 (PGE2), which attracts MDSC into the ascites microenvironment. MDSCs migrated toward ascites in a CXCR4-dependent manner that required COX2 activity and autocrine PGE2 production [5]. Two years later, another group showed that tumor-infiltrating CD33^+^ MDSCs were significantly associated with shorter overall survival (OS) and a reduced disease-free interval (DFI). Functionally, Cui et al. demonstrated the interaction between MDSCs and CSCs in ovarian cancer patients and showed that MDSCs inhibited T cell activation and enhanced CSC gene expression, sphere formation, and cancer metastasis [50] (Figure 2).

In 2017, three independent research groups further elucidated the role of MDSCs in ovarian cancer. First, Horikawa et al. showed that high abundance of omental CD33^+^ MDSCs was associated with worse survival in patients. The group demonstrated that VEGF expression in ovarian cancer induced the VEGFR2-mediated recruitment and differentiation of MDSCs into tumors. High MDSC infiltration was inversely correlated with the intratumoral infiltration of CD8^+^ T-cells. Functionally, MDSCs from patients’ ascites inhibited T-cell proliferation [54]. Second, Wu et al. demonstrated that ascites-derived IL-6 and IL-10 synergistically expand CD14^+^HLA-DR^−/low^ M-MDSCs in patients, and high levels of ascites and blood-derived MDSCs were associated with poor prognosis. Mechanistically, ascitic-driven STAT3 activation upregulated the expression of arginase (ARG1) and inducible nitric oxide synthase (iNOS) in M-MDSC, through which these MDSCs executed the immunosuppressive activity [55]. Third, Rodriguez-Ubreva et al. demonstrated that the in vitro differentiation of DCs from human primary monocytes results in the generation of immunosuppressive MDSCs under tumor-associated conditions including PGE2 or tumor-cell-conditioned media. MDSCs isolated from patients display a similar hypermethylation signature in connection with PGE2-dependent DNMT3A overexpression. In this study the authors link PGE2/DNMT3A-dependent hypermethylation to immunosuppressive MDSC functions [56]. These findings indicate that TME-derived factors, VEGF, IL-6, IL-10, and PGE2 can promote development and differentiation and act as chemoattractants in the recruitment of MDSCs into the TME. Further examination of soluble markers that drive MDSCs into the TME is needed (Figure 2).

In the next study, Santegoets et al. discuss the clinical aspects of different myeloid populations, showing the M-MDSC to DC ratio as an independent, predictive factor for survival. Additionally, they revealed that high levels of circulating myeloid cells are associated with poor survival after therapy. Functionally, patients’ M-MDSCs were shown to suppress T cell reactivity in vitro [57].

In 2019, our group demonstrated the presence of three MDSC subsets, including M-MDSCs, PMN-MDSCs, and eMDSCs in three paired environments, i.e., peripheral blood, ascites, and tumor tissue, identifying an abundance of M-MDSCs in all three examined environments in the patients compared to the control group. We revealed selectively that M-MDSCs—not PMN-MDSCs and eMDSCs—were associated with worse survival [58]. In the same year Lee at el. showed that patients with BRCA mutations may have fewer circulating MDSCs but higher CD8^+^ T cells in PBMCs during their early disease course compared to BRCA wild-type ovarian cancer. Next, Coosemans’ group presented evidence that MDSCs at diagnosis may discriminate between benign and malignant ovarian tumors. Collectively, these results indicate the clinical relevance of MDSCs in ovarian cancer.

**Figure 3:**
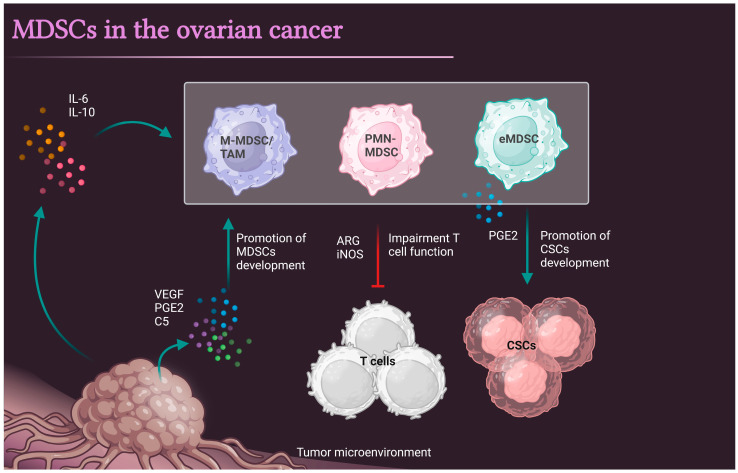
MDSCs in the pathogenesis of ovarian cancer. The accumulation of IL-6, IL-10, VEGF, PGE2, and complement C5 that occur in the TME promotes the development, accumulation and persistence of MDSCs. In the TME, MDSCs inhibit T cell accumulation and impair their functional activity. MDSCs also show potency to promote cancer stem cells (CSCs) activity.

In 2020, Li et al. showed that MDSCs promote ovarian cancer cell stemness by inducing the CSF2/p-STAT3 signaling pathway [59]. While Komura’s group indicated that MDSCs increase cancer stem-like cells and promote PD-L1 expression in ovarian cancer. In vitro co-culture of MDSCs and CSCs revealed that MDSCs increased the number of CSCs via the production of PGE2 (Figure 3) [53].

In mouse models of ovarian cancer, the metabolic characteristics of immature mouse CD11b^+^Gr1^+^ myeloid cells were presented by Udumula et al., indicating increased oxidative phosphorylation fueled by glutamine after priming by ovarian ID8 tumors [19]. The TME-enriched complement C5 promotes MDSCs’ infiltration and development in the TME to facilitate metastasis [60] (Figure 3). Yet, these findings need to be further confirmed in human MDSCs.

Because a characteristic feature of advanced-stage ovarian cancer is the accumulation of fluid (ascites) in the abdomen, which comprises cellular and acellular components, MDSCs can be highly influential cells in promoting CTCs’ spread in the peritoneal cavity and to other tissues. Indeed, it has been shown that multicellular aggregates (spheroids) accumulate with metastatic potential in the fresh ascites of ovarian cancer patients [61]. Moreover, M-MDSC rapidly differentiate into tumor-associated macrophages (TAMs) in the TME [62]. Consistent with this, it has been shown that TAMs promote spheroid formation and tumor growth at early stages of metastasis in an established mouse model for ovarian cancer. TAMs were localized in the center of spheroids and secreted EGF, which upregulated the αMβ2 integrin on TAMs and ICAM-1 on tumor cells to promote association between tumor cells and TAMs. Pharmacological blockade of EGFR or antibody neutralization of ICAM-1 in TAMs inhibited spheroid formation and ovarian cancer progression in mouse models [63]. Further studies in cancer patients are needed to determine whether the targeting of MDSCs could result in the inhibition of early stages of metastasis, serving as an effective antitumor therapeutic response.

**Table 2:** MDSCs in ovarian cancer patients.

Populations	Clinical Relevance	Ref.
CD11b^+^CD14^+^CD33^+^CXCR4^+^	ND	[5]
Lin^−^CD45^+^CD33^+^	High level is associated with poor OS	[50]
CD33^+^	High level is associated with poor OS	[54]
CD14^+^HLA-DR^−/low^	High level is associated with shorter RFS	[55]
CD11b^+^CD14^+^CD15^−^ M-MDSCs	ND	[56]
CD33^+^	ND	[64]
CD3^−^CD19^−^CD56^−^HLA-DR^−/low^CD14^+^CD15^−^ M-MDSCs CD3^−^CD19^−^CD56^−^HLA-DR^−/low^CD14^−^CD15^−^ and CD33^+^CD11b^+^ early stage eMDSCs	Circulating MDSCs are associated with poor survival after therapy Low DC/M-MDSC ratio is associated with poor OS	[57]
CD3^−^CD19^−^CD56^−^HLA-DR^−/low^ and CD14^–^CD15^–^ double-negative (dn) CD33^−^CD11b^+^ MDSC (CD33^−^ dnMDSCs).		
HLA-DR^−/low^CD11b^+^CD14^+^CD15^−^M-MDSCs HLA-DR^−/low^CD11b^+^CD14^−^CD15^+^ PMN-MDSCs	High level of M-MDSCs is associated with poor OS	[58]
HLA-DR^−/low^CD11b^+^Lin^−^CD33^+^ eMDSCs		
M-MDSCs, PMN-MDSCs, Lin^−^ MDSCs	BRCA mutations was associated with decreased MDSCs	[65]
M-MDSCs and PMN-MDSCs	Increased MDSCs was found to be an independent predictor of malignancy	[66]
M-MDSCs and PMN-MDSCs	ND	[59]
CD33^+^	ND	[53]

ND—not determined.

## 4. Therapeutic Application of MDSCs

The continuous recruitment of MDSCs enables them to have long-lasting effects in the TME and promote tumor persistence. A few strategies to target MDSCs have been proposed in preclinical mouse OC models [67], including anti-GR-1 antibodies [53,54], anti-GM-CSF antibodies [68], CXCR2/4 antagonists [64,69], PGE2/COX-2 inhibition [5], metformin [70], thrombin inhibitors [71], and bis-benzylidine piperidone RA190 [72]. These agents showed significant antitumor efficacy when used as monotherapies or in combination with chemotherapy. Furthermore, it has been shown that MDSC-inhibition therapies targeting CXCR4 and IL-10 enhance the therapeutic efficacy of anti-PD-1 treatment, thereby leading to prolonged survival [73,74]. However, until now, there have been no clinical trials targeting MDSCs in ovarian cancer patients. Due to their short-life span in tissues, the state of pathological activation of these cells in the TME is difficult to reverse as they quickly differentiate into TAMs in the TME. Nevertheless, effective therapies could be implemented to target MDSCs by blocking their migration to the TME and immunosuppressive functions through the inhibition of VEGF, PGE2, IL-6, IL-10, component C5, ARG, and iNOS. Secondary options include the depletion of CD33^+^ myeloid populations, known to promote ovarian CSCs and inhibit CD8^+^ T cell function. As in ovarian cancer metastasis, with the major role played by the local TME, including MDSCs-abundant ascitic fluid, the treatment efficacy could be optimized by using the local delivery of MDSC-targeting agents, which should be evaluated in future clinical trials. Third, studies have shown the metabolic (glutamine) dependence of the immunosuppressive function of myeloid cells mediated by the ovarian TME [19], thus inhibiting the glutamine pathway which can be of clinical importance. Finally, as MDSCs differentiate into TAMs in the TME, a better option would be targeting TAMs, which constitute up to 50% of the cell mass within the TME of most solid tumors [75]. When combined with cyclophosphamide (CPA), a new therapeutic approach based on human engineered macrophages modified to release human cytochrome P450 was effectively explored for the in vivo treatment of the ovarian PDX model. In order to express cytochrome P450, genetically modified macrophages generated from human monocytes were infected with adenoviral particles. Engineered macrophages located in the TME then released P450 to transform CPA into hazardous metabolites, which caused cancer cell death in ovarian PDX mice. This led to a two-fold increase in overall survival [76]. A novel approach also includes the engineered reprogramming of TAMs [77] by using chimeric antigen receptor (CAR)-engineered macrophages [78]. The anti-HER2 CAR-M from Klichinsky and collaborators successfully demonstrated a reduction in ovarian cancer tumor burden in mouse models and was evaluated in a first-in-human phase 1 clinical trial that focused on patients with recurrent or metastatic HER-2-overexpressing solid tumors, including ovarian cancer (NCT04660929) [79].

## 5. Perspectives

OC is a highly deadly form of cancer with poor responsiveness to the existing immunotherapies. MDSCs exhibit several mechanisms to evade the immune response and promote the aggressiveness of OC. High-throughput technologies, e.g., single-cell RNA sequencing (scRNAseq) analysis, possess great potential for exploring MDSC signatures involved in tumor development and progression in ovarian cancer. A few studies have already shown different transcriptomic profiles of myeloid populations in human ovarian cancer [80,81]. In accordance with the results described above, the scRNAseq analysis of tumor tissues suggests dynamic plasticity and transformation among M1-like, MDSC, and M2-like macrophages in the TME of HGSOC tumors [7]. Further studies will be needed to elucidate the dynamic nature of MDSCs/TAMs in the TME of ovarian cancer, which will help in the development of strategies aimed at therapeutically targeting these cells.

Engineered reprogramming of myeloid cells using CAR-M [79] may become a promising anti-cancer strategy, yet a few challenges remain. First, myeloid cells are extremely plastic and can adapt to their phenotype and function in response to TME stimuli. Second, the limited expansion and persistence of CAR-M in vivo with current technology may obstruct therapeutic efficacy. Third, limiting toxicity and immunogenicity should also be considered when developing CAR-M technology. Strategies for new-generation CAR-M should include specific tumor antigen selection, improved expansion and persistence, feasible genetic modification, and the control of safety. A recent study showed the in vivo generation of mRNA-based CAR T cells to eliminate activated fibroblasts [82]. Therefore, the generation of mRNA-based CAR T cells to eliminate MDSCs/TAMs can be beneficial as a means of promoting rapid protection against these cells. Next, strategies can use engineered primary myeloid cells to produce pro-inflammatory substances to attract and activate anti-cancer immune cells within the TME, inhibit the expression of the genes responsible for immunosuppression, enhance phagocytosis, release anti-cancer drugs, or deliver chemotherapies [83].

The infiltration of myeloid populations can be a major barrier to an effective (immuno)therapeutic response in OC, as this population of cells promotes the exclusion of T cells in the TME. It has been shown that MDSCs are associated with resistance to anti-PD-1 therapy [84]. Moreover, the immunosuppressive TME in ascites, which contains myeloid cells, has a role in both recurrence and chemoresistance in ovarian cancer [7]. All-trans retinoic acid (ATRA) may induce the maturation of MDSCs and alter their immunosuppressive activity. Adding ATRA to pembrolizumab may target this resistance mechanism to enhance the overall impact of anti-PD-1-based immunotherapy [84]. Similarly, vitamin D signaling has also been reported to decrease the immunosuppressive capabilities of MDSCs through the vitamin D receptor (VDR) [81]. Whether inhibition of MDSCs by using ATRA or vitamin D can sensitize ovarian tumors to chemotherapy and/or immunotherapy is unknown. However, anti-TREM2 mAbs were demonstrated in preclinical ovarian cancer models to deplete TAMs, enhance intratumoral CD8^+^ T cell activation, and reverse anti-PD-1 treatment resistance. Humanized anti-TREM2 mAb (PY314) is presently being examined in a phase I clinical trial in patients with advanced solid tumors, including OC (NCT04691375) [85].

Collectively, given all of these results and emerging new technologies, we may better understand the nature of MDSCs and further use our knowledge to design more effective, next-generation immunotherapeutic strategies for ovarian cancer.

## Figures and Tables

**Table 1:** MDSC-mediated tumor-promoting effects.

MDSC-Mediated Suppression		
Immunosuppressive Functions of MDSCs		
Expression of immune checkpoint inhibitors	↑ PD-L1 expression induces T-cell anergy	[23,24]
	↑ CTLA-4 expression	[25]
	↑ VISTA expression is associated with PD-1^+^ T cells	[26]
	↑ Gal-9 expression suppresses T cell responses	[27,28]
	↑ CD155 expression is associated with T cell inhibition	[29]
Depletion of nutrients	↑ ARG1 release is associated with T cells’ inhibition	[30]
	↑ Methylglyoxal induces T cell suppression	[18]
	↓ Tryptophan induces T cell autophagy, cell cycle arrest, and death	[31]
	↓ Cysteine is associated with the impairment of T cell activation	[32]
Promotion of oxidative stress	↑ ROS catalyzes the nitration of TCR/CD8 molecules	[33]
	↑ RNS reduces the affinity of CCL2 to CCR2 which inhibits TILs’ recruitment	[34]
	↑ iNOS inhibits T cells	[35,36]
Inhibition of T cell trafficking	M-MDSCs-derived NO damages T cells’ extravasation and tissue infiltration by the downregulation of CD44 and CD162 on T cells	[37]
	ADAM17 expressed on MDSCs cleaves the CD62L on naive T cells to inhibit their trafficking to peripheral lymph nodes and the tumor niche	[38]
Crosstalk between MDSCs and other immune cells	M-MDSCs promote NK cells anergy	[39]
	PMN-MDSCs block the antigen cross-presentation of dendritic cells by transferring oxidized lipids	[40]
	MDSCs inhibit B cells by modulating the IL-7 and STAT5 pathways	[41]
	MDSCs promote PD-L1 expression on B cells	[42]
	M-MDSCs produce CCR5 ligands to chemoattract Tregs	[43]
	MDSCs induce Tregs through the secretion of IL-10 and TGF-β or/and the expression of ARG1, IDO, and CD40	[44,45]
	MDSCs elicit a type 2 tumor-promoting immune response, which is mediated by elevated IL-10 and downregulated IL-12 production	[46]
**Non-immunosuppressive functions of MDSCs**		
Promotion of angiogenesis	Secretion of soluble interleukins, CCL2, CXCL2, BV8, and MMPs	[47]
	Secretion of exosomes which release proangiogenic factors	[48]
Promotion of stemness of tumor cells, facilitating epithelial–mesenchymal transition and pre-metastatic niche formation	PMN-MDSCs-derived exosomal S100A9 promotes cancer stemness in a HIF-1α-dependent manner	[49]
	MDSCs promote miRNA101 expression and repress CtBP2 in cancer cells, leading to increased cancer cell stemness and metastatic potential	[50]
	M-MDSCs promote the EMT/CSC phenotype by facilitating tumor cell dissemination.	[51]
	↑ IL-6 activates the STAT3-mediated stem-like properties of cancer cells	[52]
	↑ PGE2 increases the stem-like properties of cancer cells	[53]

## References

[1] Gabrilovich D.I., Bronte V., Chen S.-H., Colombo M.P., Ochoa A., Ostrand-Rosenberg S., Schreiber H. (2007). The Terminology Issue for Myeloid-Derived Suppressor Cells. Cancer Res..

[2] Talmadge J.E., Gabrilovich D.I. (2013). History of Myeloid Derived Suppressor Cells (MDSCs) in the Macro- and Micro-Environment of Tumour-Bearing Hosts. Nat. Rev. Cancer.

[3] Marvel D., Gabrilovich D.I. (2015). Myeloid-Derived Suppressor Cells in the Tumor Microenvironment: Expect the Unexpected. J. Clin. Investig..

[4] Ugel S., Sanctis F.D., Mandruzzato S., Bronte V. (2015). Tumor-Induced Myeloid Deviation: When Myeloid-Derived Suppressor Cells Meet Tumor-Associated Macrophages. J. Clin. Investig..

[5] Obermajer N., Muthuswamy R., Odunsi K., Edwards R.P., Kalinski P. (2011). PGE2-Induced CXCL12 Production and CXCR4 Expression Controls the Accumulation of Human MDSCs in Ovarian Cancer Environment. Cancer Res..

[6] Siegel R.L., Miller K.D., Jemal A. (2019). Cancer Statistics, 2019. CA A Cancer J. Clin..

[7] Xu J., Fang Y., Chen K., Li S., Tang S., Ren Y., Cen Y., Fei W., Zhang B., Shen Y. (2022). Single-Cell RNA Sequencing Reveals the Tissue Architecture in Human High-Grade Serous Ovarian Cancer. Clin. Cancer Res..

[8] Chardin L., Leary A. (2021). Immunotherapy in Ovarian Cancer: Thinking Beyond PD-1/PD-L1. Front. Oncol..

[9] Fucikova J., Coosemans A., Orsulic S., Cibula D., Vergote I., Galluzzi L., Spisek R. (2021). Immunological Configuration of Ovarian Carcinoma: Features and Impact on Disease Outcome. J. Immunother. Cancer.

[10] Vonderheide R.H., Bear A.S. (2020). Tumor-Derived Myeloid Cell Chemoattractants and T Cell Exclusion in Pancreatic Cancer. Front. Immunol..

[11] Gabrilovich D.I. (2017). Myeloid-Derived Suppressor Cells. Cancer Immunol. Res..

[12] Veglia F., Sanseviero E., Gabrilovich D.I. (2021). Myeloid-Derived Suppressor Cells in the Era of Increasing Myeloid Cell Diversity. Nat. Rev. Immunol..

[13] Tellez R.S.L., Reynolds L., Piris M.A. (2023). Myeloid-Derived Suppressor Cells (MDSCs): What Do We Currently Know about the Effect They Have against Anti-PD-1/PD-L1 Therapies?. Ecancermedicalscience.

[14] Lim H.X., Kim T.S., Poh C.L. (2020). Understanding the Differentiation, Expansion, Recruitment and Suppressive Activities of Myeloid-Derived Suppressor Cells in Cancers. Int. J. Mol. Sci..

[15] Bader J.E., Voss K., Rathmell J.C. (2020). Targeting Metabolism to Improve the Tumor Microenvironment for Cancer Immunotherapy. Mol. Cell.

[16] Yan D., Yang Q., Shi M., Zhong L., Wu C., Meng T., Yin H., Zhou J. (2013). Polyunsaturated Fatty Acids Promote the Expansion of Myeloid-Derived Suppressor Cells by Activating the JAK/STAT3 Pathway. Eur. J. Immunol..

[17] Jian S.-L., Chen W.-W., Su Y.-C., Su Y.-W., Chuang T.-H., Hsu S.-C., Huang L.-R. (2017). Glycolysis Regulates the Expansion of Myeloid-Derived Suppressor Cells in Tumor-Bearing Hosts through Prevention of ROS-Mediated Apoptosis. Cell Death Dis..

[18] Baumann T., Dunkel A., Schmid C., Schmitt S., Hiltensperger M., Lohr K., Laketa V., Donakonda S., Ahting U., Lorenz-Depiereux B. (2020). Regulatory Myeloid Cells Paralyze T Cells through Cell–Cell Transfer of the Metabolite Methylglyoxal. Nat. Immunol..

[19] Udumula M.P., Sakr S., Dar S., Alvero A.B., Ali-Fehmi R., Abdulfatah E., Li J., Jiang J., Tang A., Buekers T. (2021). Ovarian Cancer Modulates the Immunosuppressive Function of CD11b+Gr1+ Myeloid Cells via Glutamine Metabolism. Mol. Metab..

[20] Li K., Shi H., Zhang B., Ou X., Ma Q., Chen Y., Shu P., Li D., Wang Y. (2021). Myeloid-Derived Suppressor Cells as Immunosuppressive Regulators and Therapeutic Targets in Cancer. Signal Transduct. Target. Ther..

[21] Ghalehbandi S., Yuzugulen J., Pranjol M.Z.I., Pourgholami M.H. (2023). The Role of VEGF in Cancer-Induced Angiogenesis and Research Progress of Drugs Targeting VEGF. Eur. J. Pharmacol..

[22] Seyfried T.N., Huysentruyt L.C. (2013). On the Origin of Cancer Metastasis. Crit. Rev. Oncog..

[23] Noman M.Z., Desantis G., Janji B., Hasmim M., Karray S., Dessen P., Bronte V., Chouaib S. (2014). PD-L1 Is a Novel Direct Target of HIF-1α, and Its Blockade under Hypoxia Enhanced MDSC-Mediated T Cell Activation. J. Exp. Med..

[24] Antonios J.P., Soto H., Everson R.G., Moughon D., Orpilla J.R., Shin N.P., Sedighim S., Treger J., Odesa S., Tucker A. (2017). Immunosuppressive Tumor-Infiltrating Myeloid Cells Mediate Adaptive Immune Resistance via a PD-1/PD-L1 Mechanism in Glioblastoma. Neuro Oncol..

[25] Gentilcore G., Pico de Yago C., Poschke I., Mao Y., Nyström M., Hansson J., Masucci G.V., Kiessling R. (2014). Ipilimumab Treatment Results in an Early Decrease in the Frequency of Circulating Granulocytic Myeloid Derived Suppressor Cells as Well as Their Arginase 1 Production. J. Transl. Med..

[26] Wang L., Jia B., Claxton D.F., Ehmann W.C., Rybka W.B., Mineishi S., Naik S., Khawaja M.R., Sivik J., Han J. (2018). VISTA Is Highly Expressed on MDSCs and Mediates an Inhibition of T Cell Response in Patients with AML. Oncoimmunology.

[27] Sakuishi K., Jayaraman P., Behar S.M., Anderson A.C., Kuchroo V.K. (2011). Emerging Tim-3 Functions in Anti-Microbial and Tumor Immunity. Trends Immunol..

[28] Limagne E., Richard C., Thibaudin M., Fumet J.-D., Truntzer C., Lagrange A., Favier L., Coudert B., Ghiringhelli F. (2019). Tim-3/Galectin-9 Pathway and MMDSC Control Primary and Secondary Resistances to PD-1 Blockade in Lung Cancer Patients. Oncoimmunology.

[29] Wu L., Mao L., Liu J.-F., Chen L., Yu G.-T., Yang L.-L., Wu H., Bu L.-L., Kulkarni A.B., Zhang W.-F. (2019). Blockade of TIGIT/CD155 Signaling Reverses T-Cell Exhaustion and Enhances Antitumor Capability in Head and Neck Squamous Cell Carcinoma. Cancer Immunol. Res..

[30] Arginase I–Producing Myeloid-Derived Suppressor Cells in Renal Cell Carcinoma Are a Subpopulation of Activated Granulocytes—PMC. https://www.ncbi.nlm.nih.gov/pmc/articles/PMC2900845/.

[31] Yu J., Du W., Yan F., Wang Y., Li H., Cao S., Yu W., Shen C., Liu J., Ren X. (2013). Myeloid-Derived Suppressor Cells Suppress Antitumor Immune Responses through IDO Expression and Correlate with Lymph Node Metastasis in Patients with Breast Cancer. J. Immunol..

[32] Srivastava M.K., Sinha P., Clements V.K., Rodriguez P., Ostrand-Rosenberg S. (2010). Myeloid-Derived Suppressor Cells Inhibit T-Cell Activation by Depleting Cystine and Cysteine. Cancer Res..

[33] Mechanism Regulating Reactive Oxygen Species in Tumor Induced Myeloid-Derived Suppressor Cells—PMC. https://www.ncbi.nlm.nih.gov/pmc/articles/PMC2833019/.

[34] Molon B., Ugel S., Del Pozzo F., Soldani C., Zilio S., Avella D., De Palma A., Mauri P., Monegal A., Rescigno M. (2011). Chemokine Nitration Prevents Intratumoral Infiltration of Antigen-Specific T Cells. J. Exp. Med..

[35] Myeloid Suppressor Lines Inhibit T Cell Responses by an NO-Dependent Mechanism1|The Journal of Immunology|American Association of Immunologists. https://journals.aai.org/jimmunol/article/168/2/689/33670/Myeloid-Suppressor-Lines-Inhibit-T-Cell-Responses.

[36] Nagaraj S., Schrum A.G., Cho H.-I., Celis E., Gabrilovich D.I. (2010). Mechanism of T-Cell Tolerance Induced by Myeloid-Derived Suppressor Cells. J. Immunol..

[37] Schouppe E., Mommer C., Movahedi K., Laoui D., Morias Y., Gysemans C., Luyckx A., De Baetselier P., Van Ginderachter J.A. (2013). Tumor-Induced Myeloid-Derived Suppressor Cell Subsets Exert either Inhibitory or Stimulatory Effects on Distinct CD8+ T-Cell Activation Events. Eur. J. Immunol..

[38] Hanson E.M., Clements V.K., Sinha P., Ilkovitch D., Ostrand-Rosenberg S. (2009). Myeloid-Derived Suppressor Cells Down-Regulate L-Selectin Expression on CD4+ and CD8+ T Cells. J. Immunol..

[39] Hoechst B., Voigtlaender T., Ormandy L., Gamrekelashvili J., Zhao F., Wedemeyer H., Lehner F., Manns M.P., Greten T.F., Korangy F. (2009). Myeloid Derived Suppressor Cells Inhibit Natural Killer Cells in Patients with Hepatocellular Carcinoma via the NKp30 Receptor. Hepatology.

[40] JCI Insight—Polymorphonuclear Myeloid-Derived Suppressor Cells Limit Antigen Cross-Presentation by Dendritic Cells in Cancer. https://insight.jci.org/articles/view/138581.

[41] Wang Y., Schafer C.C., Hough K.P., Tousif S., Duncan S.R., Kearney J.F., Ponnazhagan S., Hsu H.-C., Deshane J.S. (2018). Myeloid-Derived Suppressor Cells Impair B Cell Responses in Lung Cancer through IL-7 and STAT5. J. Immunol..

[42] Shen M., Wang J., Yu W., Zhang C., Liu M., Wang K., Yang L., Wei F., Wang S.E., Sun Q. (2018). A Novel MDSC-Induced PD-1−PD-L1+ B-Cell Subset in Breast Tumor Microenvironment Possesses Immuno-Suppressive Properties. Oncoimmunology.

[43] Schlecker E., Stojanovic A., Eisen C., Quack C., Falk C.S., Umansky V., Cerwenka A. (2012). Tumor-Infiltrating Monocytic Myeloid-Derived Suppressor Cells Mediate CCR5-Dependent Recruitment of Regulatory T Cells Favoring Tumor Growth. J. Immunol..

[44] Siret C., Collignon A., Silvy F., Robert S., Cheyrol T., André P., Rigot V., Iovanna J., van de Pavert S., Lombardo D. (2020). Deciphering the Crosstalk Between Myeloid-Derived Suppressor Cells and Regulatory T Cells in Pancreatic Ductal Adenocarcinoma. Front. Immunol..

[45] Cancers | Free Full-Text | The Functional Crosstalk between Myeloid-Derived Suppressor Cells and Regulatory T Cells within the Immunosuppressive Tumor Microenvironment. https://www.mdpi.com/2072-6694/13/2/210.

[46] Ostrand-Rosenberg S., Sinha P., Beury D.W., Clements V.K. (2012). Cross-Talk between Myeloid-Derived Suppressor Cells (MDSC), Macrophages, and Dendritic Cells Enhances Tumor-Induced Immune Suppression. Semin. Cancer Biol..

[47] Albini A., Bruno A., Noonan D.M., Mortara L. (2018). Contribution to Tumor Angiogenesis From Innate Immune Cells Within the Tumor Microenvironment: Implications for Immunotherapy. Front. Immunol..

[48] Deng Z., Rong Y., Teng Y., Zhuang X., Samykutty A., Mu J., Zhang L., Cao P., Yan J., Miller D. (2017). Exosomes MiR-126a Released from MDSC Induced by DOX Treatment Promotes Lung Metastasis. Oncogene.

[49] Wang Y., Yin K., Tian J., Xia X., Ma J., Tang X., Xu H., Wang S. (2019). Granulocytic Myeloid-Derived Suppressor Cells Promote the Stemness of Colorectal Cancer Cells through Exosomal S100A9. Adv. Sci. Weinh. Baden-Wurtt. Ger..

[50] Cui T.X., Kryczek I., Zhao L., Zhao E., Kuick R., Roh M.H., Vatan L., Szeliga W., Mao Y., Thomas D.G. (2013). Myeloid Derived Suppressor Cells Enhance Stemness of Cancer Cells by Inducing MicroRNA101 and Suppressing the Corepressor CtBP2. Immunity.

[51] Bayik D., Zhou Y., Park C., Hong C., Vail D., Silver D.J., Lauko A., Roversi G., Watson D.C., Lo A. (2020). Myeloid-Derived Suppressor Cell Subsets Drive Glioblastoma Growth in a Sex-Specific Manner. Cancer Discov..

[52] Peng D., Tanikawa T., Li W., Zhao L., Vatan L., Szeliga W., Wan S., Wei S., Wang Y., Liu Y. (2016). Myeloid-Derived Suppressor Cells Endow Stem-like Qualities to Breast Cancer Cells through IL6/STAT3 and NO/NOTCH Cross-talk Signaling. Cancer Res..

[53] Komura N., Mabuchi S., Shimura K., Yokoi E., Kozasa K., Kuroda H., Takahashi R., Sasano T., Kawano M., Matsumoto Y. (2020). The Role of Myeloid-Derived Suppressor Cells in Increasing Cancer Stem-Like Cells and Promoting PD-L1 Expression in Epithelial Ovarian Cancer. Cancer Immunol. Immunother..

[54] Horikawa N., Abiko K., Matsumura N., Hamanishi J., Baba T., Yamaguchi K., Yoshioka Y., Koshiyama M., Konishi I. (2017). Expression of Vascular Endothelial Growth Factor in Ovarian Cancer Inhibits Tumor Immunity through the Accumulation of Myeloid-Derived Suppressor Cells. Clin. Cancer Res..

[55] Wu L., Deng Z., Peng Y., Han L., Liu J., Wang L., Li B., Zhao J., Jiao S., Wei H. (2017). Ascites-Derived IL-6 and IL-10 Synergistically Expand CD14+HLA-DR-/Low Myeloid-Derived Suppressor Cells in Ovarian Cancer Patients. Oncotarget.

[56] Rodríguez-Ubreva J., Català-Moll F., Obermajer N., Álvarez-Errico D., Ramirez R.N., Company C., Vento-Tormo R., Moreno-Bueno G., Edwards R.P., Mortazavi A. (2017). Prostaglandin E2 Leads to the Acquisition of DNMT3A-Dependent Tolerogenic Functions in Human Myeloid-Derived Suppressor Cells. Cell Rep..

[57] Santegoets S.J.A.M., de Groot A.F., Dijkgraaf E.M., Simões A.M.C., van der Noord V.E., van Ham J.J., Welters M.J.P., Kroep J.R., van der Burg S.H. (2018). The Blood MMDSC to DC Ratio Is a Sensitive and Easy to Assess Independent Predictive Factor for Epithelial Ovarian Cancer Survival. Oncoimmunology.

[58] Okła K., Czerwonka A., Wawruszak A., Bobiński M., Bilska M., Tarkowski R., Bednarek W., Wertel I., Kotarski J. (2019). Clinical Relevance and Immunosuppressive Pattern of Circulating and Infiltrating Subsets of Myeloid-Derived Suppressor Cells (MDSCs) in Epithelial Ovarian Cancer. Front. Immunol..

[59] Li X., Wang J., Wu W., Gao H., Liu N., Zhan G., Li L., Han L., Guo X. (2020). Myeloid-derived Suppressor Cells Promote Epithelial Ovarian Cancer Cell Stemness by Inducing the CSF2/P-STAT3 Signalling Pathway. FEBS J..

[60] Li Y., Zhang Q., Wu M., Zhang P., Huang L., Ai X., Yang Z., Shen Q., Wang Y., Wang P. (2022). Suppressing MDSC Infiltration in Tumor Microenvironment Serves as an Option for Treating Ovarian Cancer Metastasis. Int. J. Biol. Sci..

[61] Velletri T., Villa C.E., Cilli D., Barzaghi B., Lo Riso P., Lupia M., Luongo R., López-Tobón A., De Simone M., Bonnal R.J.P. (2022). Single Cell-Derived Spheroids Capture the Self-Renewing Subpopulations of Metastatic Ovarian Cancer. Cell Death Differ..

[62] Kumar V., Cheng P., Condamine T., Mony S., Languino L., McCaffrey J., Hockstein N., Guarino M., Masters G., Penman E. (2016). CD45 Phosphatase Regulates the Fate of Myeloid Cells in Tumor Microenvironment by Inhibiting STAT3 Activity. J. Immunol..

[63] Yin M., Li X., Tan S., Zhou H.J., Ji W., Bellone S., Xu X., Zhang H., Santin A.D., Lou G. (2016). Tumor-Associated Macrophages Drive Spheroid Formation during Early Transcoelomic Metastasis of Ovarian Cancer. J. Clin. Investig..

[64] Taki M., Abiko K., Baba T., Hamanishi J., Yamaguchi K., Murakami R., Yamanoi K., Horikawa N., Hosoe Y., Nakamura E. (2018). Snail Promotes Ovarian Cancer Progression by Recruiting Myeloid-Derived Suppressor Cells via CXCR2 Ligand Upregulation. Nat. Commun..

[65] Lee J.-M., Botesteanu D.-A., Tomita Y., Yuno A., Lee M.-J., Kohn E.C., Annunziata C.M., Matulonis U., MacDonald L.A., Nair J.R. (2019). Patients with BRCA Mutated Ovarian Cancer May Have Fewer Circulating MDSC and More Peripheral CD8+ T Cells Compared with Women with BRCA Wild-Type Disease during the Early Disease Course. Oncol. Lett..

[66] Coosemans A., Baert T., Ceusters J., Busschaert P., Landolfo C., Verschuere T., Van Rompuy A.-S., Vanderstichele A., Froyman W., Neven P. (2019). Myeloid-Derived Suppressor Cells at Diagnosis May Discriminate between Benign and Malignant Ovarian Tumors. Int. J. Gynecol. Cancer.

[67] Mabuchi S., Sasano T., Komura N. (2021). Targeting Myeloid-Derived Suppressor Cells in Ovarian Cancer. Cells.

[68] Horikawa N., Abiko K., Matsumura N., Baba T., Hamanishi J., Yamaguchi K., Murakami R., Taki M., Ukita M., Hosoe Y. (2020). Anti-VEGF Therapy Resistance in Ovarian Cancer Is Caused by GM-CSF-Induced Myeloid-Derived Suppressor Cell Recruitment. Br. J. Cancer.

[69] Zeng Y., Li B., Liang Y., Reeves P.M., Qu X., Ran C., Liu Q., Callahan M.V., Sluder A.E., Gelfand J.A. (2019). Dual Blockade of CXCL12-CXCR4 and PD-1–PD-L1 Pathways Prolongs Survival of Ovarian Tumor–Bearing Mice by Prevention of Immunosuppression in the Tumor Microenvironment. FASEB J..

[70] Li L., Wang L., Li J., Fan Z., Yang L., Zhang Z., Zhang C., Yue D., Qin G., Zhang T. (2018). Metformin-Induced Reduction of CD39 and CD73 Blocks Myeloid-Derived Suppressor Cell Activity in Patients with Ovarian Cancer. Cancer Res..

[71] Soong R.-S., Anchoori R.K., Yang B., Yang A., Tseng S.-H., He L., Tsai Y.-C., Roden R.B.S., Hung C.-F. (2016). RPN13/ADRM1 Inhibitor Reverses Immunosuppression by Myeloid-Derived Suppressor Cells. Oncotarget.

[72] Alexander E.T., Minton A.R., Peters M.C., van Ryn J., Gilmour S.K. (2016). Thrombin Inhibition and Cisplatin Block Tumor Progression in Ovarian Cancer by Alleviating the Immunosuppressive Microenvironment. Oncotarget.

[73] Baert T., Vankerckhoven A., Riva M., Van Hoylandt A., Thirion G., Holger G., Mathivet T., Vergote I., Coosemans A. (2019). Myeloid Derived Suppressor Cells: Key Drivers of Immunosuppression in Ovarian Cancer. Front. Immunol..

[74] Lamichhane P., Karyampudi L., Shreeder B., Krempski J., Bahr D., Daum J., Kalli K.R., Goode E.L., Block M.S., Cannon M.J. (2017). IL-10 Release upon PD-1 Blockade Sustains Immunosuppression in Ovarian Cancer. Cancer Res..

[75] Wang S., Yang Y., Ma P., Zha Y., Zhang J., Lei A., Li N. (2022). CAR-Macrophage: An Extensive Immune Enhancer to Fight Cancer. eBioMedicine.

[76] Kan O., Day D., Iqball S., Burke F., Grimshaw M.J., Naylor S., Binley K. (2011). Genetically Modified Macrophages Expressing Hypoxia Regulated Cytochrome P450 and P450 Reductase for the Treatment of Cancer. Int. J. Mol. Med..

[77] Villanueva M.T. (2020). Macrophages Get a CAR. Nat. Rev. Immunol..

[78] Su S., Lei A., Wang X., Lu H., Wang S., Yang Y., Li N., Zhang Y., Zhang J. (2022). Induced CAR-Macrophages as a Novel Therapeutic Cell Type for Cancer Immune Cell Therapies. Cells.

[79] Klichinsky M., Ruella M., Shestova O., Lu X.M., Best A., Zeeman M., Schmierer M., Gabrusiewicz K., Anderson N.R., Petty N.E. (2020). Human Chimeric Antigen Receptor Macrophages for Cancer Immunotherapy. Nat. Biotechnol..

[80] Izar B., Tirosh I., Stover E.H., Wakiro I., Cuoco M.S., Alter I., Rodman C., Leeson R., Su M.-J., Shah P. (2020). A Single-Cell Landscape of High-Grade Serous Ovarian Cancer. Nat. Med..

[81] Zhang K., Erkan E.P., Jamalzadeh S., Dai J., Andersson N., Kaipio K., Lamminen T., Mansuri N., Huhtinen K., Carpén O. (2022). Longitudinal Single-Cell RNA-Seq Analysis Reveals Stress-Promoted Chemoresistance in Metastatic Ovarian Cancer. Sci. Adv..

[82] Rurik J.G., Tombácz I., Yadegari A., Méndez Fernández P.O., Shewale S.V., Li L., Kimura T., Soliman O.Y., Papp T.E., Tam Y.K. (2022). CAR T Cells Produced in Vivo to Treat Cardiac Injury. Science.

[83] Canella A., Rajappa P. (2023). Therapeutic Utility of Engineered Myeloid Cells in the Tumor Microenvironment. Cancer Gene Ther..

[84] Olson D.J., Luke J.J. (2023). Myeloid Maturity: ATRA to Enhance Anti–PD-1?. Clin. Cancer Res..

[85] Truxova I., Cibula D., Spisek R., Fucikova J. (2023). Targeting Tumor-Associated Macrophages for Successful Immunotherapy of Ovarian Carcinoma. J. Immunother. Cancer.

